# Theoretical Modeling of Oral Glucose Tolerance Tests Guides the Interpretation of the Impact of Perinatal Cadmium Exposure on the Offspring’s Glucose Homeostasis

**DOI:** 10.3390/toxics8020030

**Published:** 2020-04-15

**Authors:** Alexandre Rocca, Eric Fanchon, Jean-Marc Moulis

**Affiliations:** 1 LJK, Grenoble INP, CNRS, Inria, Université Grenoble Alpes, 38000 Grenoble, France; 2 TIMC-IMAG, UMR 5525, CNRS, Université Grenoble Alpes, 38041 Grenoble, France; 3 Laboratory of Fundamental and Applied Bioenergetics (LBFA), CEA, Inserm U1005, Université Grenoble Alpes, 38000 Grenoble, France; jean-marc.moulis@cea.fr

**Keywords:** OGTT, minimal model, cadmium, glucose response mechanism

## Abstract

Oral glucose tolerance tests, in which the concentration of glucose is monitored in the circulation over 2 h after ingesting a bolus, probe diabetic or pre-diabetic conditions. The resulting glucose curves inform about glucose turnover, insulin production and sensitivity, and other parameters. However, extracting the relevant parameters from a single complex curve is not straightforward. We propose a simple modeling method recapitulating the most salient features of the role of insulin-secreting pancreatic β-cells and insulin sensitive tissues. This method implements four ordinary differential equations with ten parameters describing the time-dependence of glucose concentration, its removal rate, and the circulating and stored insulin concentrations. From the initial parameter set adjusted to a reference condition, fitting is done by minimizing a weighted least-square residual. In doing so, the sensitivity of β-cells to glucose was identified as the most likely impacted function at weaning for the progeny of rats that were lightly exposed to cadmium in the perigestational period. Later in life, after young rats received non-contaminated carbohydrate enriched food, differences are more subtle, but modeling agrees with long-lasting perturbation of glucose homeostasis.

## 1. Introduction

The diagnostic and monitoring of diabetes mellitus rely on the experimental assessment of glucose homeostasis. Various tests and indices have been developed over time with the aim of identifying the sources of dysfunction among two main categories, deficient insulin secretion and insulin resistance. The former reflects the failure of β-cells in pancreatic islets of Langerhans to respond to increased glucose concentrations, whereas the latter corresponds to the increased inability of insulin-sensitive tissues, such as liver, muscles, and adipose tissue, to internalize glucose upon insulin stimulation together with deficient repression by insulin of hepatic glucose synthesis. Such conditions progressively develop in pre-diabetic states, and they are the hallmark of diabetes. However, discriminating between β-cells failure and insulin resistance is a challenge and involves invasive assays [1].

Among the environmental contributors to diabetes, particularly type 2 diabetes, exposure to the widespread metal cadmium has been regularly proposed. Epidemiological data promoted the idea at the beginning of the century [2], and, since then, conflicting results have bolstered debate on the issue [3,4,5,6,7,8,9,10] without reaching any clear consensus. The same statement applies to epidemiological studies considering the influence of the cadmium burden of the mother on the glucose homeostasis of both mother and child [11,12]. Besides, mechanistic investigations on the effects of cadmium on the function of the β-cells often focused on large, short-term, exposure (see [13,14] for the latest examples of such approaches) that bear little relevance to environmental conditions. Comparatively, very few studies investigated the relationship between low-level cadmium burden and impaired glucose homeostasis in relatively well defined conditions that can be implemented in the laboratory [15].

Among currently applied assays probing glucose homeostasis, oral glucose tolerance tests (OGTT) gather several advantages such as low-end staff and patient burden, integrated physiological behavior of the main contributors to glucose homeostasis, and clinically valuable information. In clinical practice, many parameters, such as 1-h or 2-h post-load glucose values that are extracted during OGTT, fasting glucose, or more indirect markers such as glycated haemoglobin, are considered in their ability to provide sensitive and cheap ways to diagnose dysglycemia and predict the development of diabetes. However, none gathers a consensus for application to all populations, or in the presence of the many confounding conditions [16]. By contrast, in research settings as with laboratory animals, the high information content of OGTT is more readily accessible since the kinetic data relative to blood glucose increase after the bolus and glucose disposal up to 2–3 h can be obtained.

The OGTT include a wealth of quantitative information that cannot always be extracted by mere examination of the curves presenting the variations of circulating glucose over time after a bolus, or even integration in the form of the area under the curve. We propose here a simple modeling and parameter analysis of such curves after cadmium exposure. The experimental data on which the present work is based were all reported before [17,18] for groups of rat pups exposed to low-level cadmium contamination through their mothers during gestation and lactation. All experimental details are available in these [17,18] and other [19] publications. In our hands [18], one of the frequently emphasized disadvantages of OGTT, namely its variability as compared to intravenous methods, has not been encountered as witnessed by the narrow spreading of measured values observed within experimental groups. This experimental advantage sets a strong basis for detailed analysis, which should allow us to focus on the variations of different parameters of glucose homeostasis obtained for these animals [18].

The purpose of the present study was to first build a simple kinetic model describing the evolution of the glucose concentration in the context of OGTT. Then, numerical simulations were run on previously obtained experimental results [17,18]. The process allowed us to test three groups of hypothesis via the sensitivity of the associated parameters as a function of cadmium exposure at three ages of pups post-weaning. These hypotheses were grouped as: (1) insulin sensitivity of glucose withdrawing tissues such as liver and muscles; (2) insulin turnover; and (3) insulin secretion by β-cells.

## 2. Materials and Methods

### 2.1. Summary of the Animal Study on Which Modeling Was Applied

**Figure 1:**
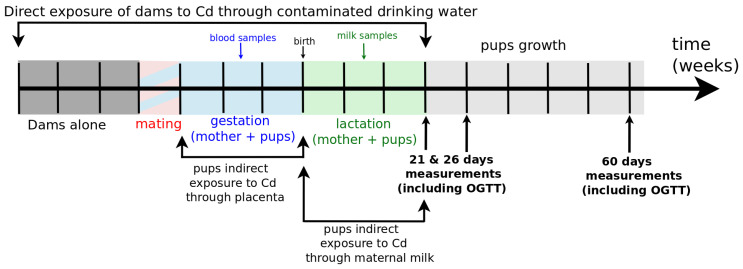
Protocol for indirect exposure of rat litters to cadmium through their mothers.

The model built in the present study was applied to previously published data on pups born from cadmium-exposed dams [18]. The animal study was approved by the ethics committee (224_LBFA-U1055, 7 April 2015) affiliated to the animal facility (D3842110001) and agreed by the French Ministry of research (approval number 02397.02, 8 January 2018). A summary of the experimental protocol is shown in Figure 1. Shortly, dams were separated into three different groups and offered ad libitum doses of cadmium (CdCl${}_{2}$) in drinking water adjusted to 0, 50, and 500 μg·(kg body mass)${}^{-1}$·day${}^{-1}$ above the diet baseline [19]. The groups were reorganized post hoc as ‘control’, Cd1, and Cd2 according to the increasing Cd concentrations of the dam’s kidneys [18]. This way, the OGTT measured for the respective pups are more representative of the cadmium exposure of the progeny via their mothers. The oral glucose tolerance tests (OGTT) measure, after overnight fasting, the evolution of plasma glucose concentration during the 2 h following force-feeding glucose intake at 2 mg per g of body mass. The tests were performed on the pups 21 days after birth, i.e., at weaning at Post-Natal Day 21 (PND21), at PND26, and at PND60. It has to be emphasized that the groups of pups were not exposed to different cadmium concentrations after weaning (>PND21) as they were all put on the same, not-intentionally cadmium-supplemented, diet. The population of the groups Control, Cd1 and Cd2 at the time points PND21, PND26, and PND60 are recalled in Table 1 to appreciate the statistical power of the studied data.

### 2.2. The Minimal Model (MINMOD)

As we plan in future work to apply formal or computationally expensive methods on our model, we built the simplest model possible while retaining important and meaningful variables for experimentalists, namely the plasma glucose concentration and the plasma insulin concentration. For this purpose, we used the minimal model (MINMOD) [20,21] as a starting point. The MINMOD model [21] is a small ODE model describing the evolution of glucose concentration after an initial intravenous injection of a glucose bolus.

(1) $$\begin{array}{cc} \dot{G} & =-p_{1}\left(G\left(t\right)-G_{b}\right)-X\left(t\right)G\left(t\right)\\ \dot{X} & =-p_{2}X\left(t\right)+p_{3}\left(I\left(t\right)-I_{b}\right)\\ \dot{I} & =-n\;I(t)+\gamma \;(G(t)-h)\;t \end{array}$$

The MINMOD model in Equation (1) has three variables: G is the glucose concentration in circulating blood, X is the rate of glucose withdrawal by muscles and adipocytes due to insulin, and I is the insulin concentration in circulating blood. This dynamic is modulated by seven parameters $p_{1},p_{2},p_{3},n,\gamma ,G_{b},I_{b}$ and *h*. Parameters $p_{1}$ is a control rate on the glucose G(t) to maintain the threshold concentration $G_{b}$ in absence of insulin regulation and glucose intake. Parameter $p_{2}$ is the decrease rate of the variable glucose absorption rate X(t). Parameter $p_{3}$ is the increase rate of X(t), and is associated to the insulin threshold $I_{b}$. The parameters associated to insulin modeling in the MINMOD model are as follows: *n* is the degradation rate of insulin and γ is the long-term insulin production rate when glucose is above threshold *h*.

### 2.3. Glucose Tolerance Test Simulation Procedures

Numerical simulations were performed in Julia using the DifferentialEquations library [22]. The process of fitting the parameter sets by minimizing Equation (5) for each dataset was performed manually. The code associated to these simulations can be found at https://github.com/roccaa/OGTT_Simulations.

## 3. Results

### 3.1. OGTT Modeling

In this approach, we first propose a model, which is adapted from the model MINMOD [21], to reproduce the OGTT results obtained in [17,18] from the protocol described in Figure 1. We refer to [23] for a review of glucose regulation models, and, together with [24,25,26,27], for a modeling of the OGTT in a more complex and exhaustive manner. Finally, we highlight the work [28] which contains a very detailed model of glucose response after a meal. The oral minimal model defined in [29] assembles three “minimal” models in order to build a quantitative minimal model of both glucose and insulin evolution in the context of OGTT. However, this model is still too complex for our simple purpose. The trade off of not using a model as detailed as [29] is that our conclusion will only be qualitative: the fitted models and parameters cannot be used for quantitative predictive purposes unlike those in [21,29].

The MINMOD model from [21] is not designed for OGTT, but for intravenous glucose tolerance tests (IvGTT). Therefore, we cannot consider that the plasma glucose is already at its maximal concentration at t = 0, as it is done for IvGTT studies.

**Figure 2:**
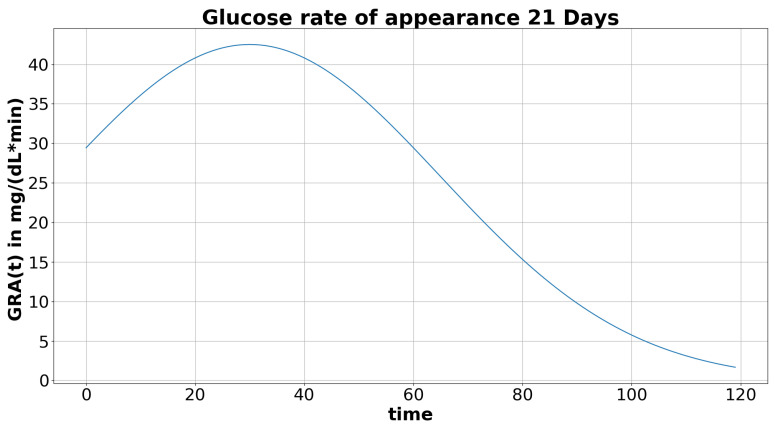
Approximation of the plasma glucose rate of appearance in 21-day-old rats (PND21).

Complex OGTT models such as [28] use compartmental modeling to represent the multiple stages of glucose distribution in the body, and to obtain the glucose rate of appearance in plasma after the meal. As a first approximation, we propose a simpler modeling using direct experimental results measuring the glucose rate of appearance after ingestion in rat, as follows. Following Wielinga et al. [30], we set the maximum of the rate of glucose appearance in the rat circulation ∼30 min after the meal (we consider that the time food spends in stomach is close to zero for the glucose solution). Similarly, the initial value of the rate of appearance was ∼70% of its maximum (see Figure 2). With this approximation, we eluded the steep increase of the glucose rate of appearance in the first few minutes of the experiments. This is justified as we focus on the simulation of the plasma glucose concentration with the first data point 10 min after glucose feeding. However, this implies imprecision with respect to the numerical variation of insulin between the fasting period and the peak of the insulin production. The curve of the glucose rate of appearance as a function of time was modeled by the continuous function $G_{\mathrm{RA}}\left(t\right)$:

(2) $$G_{\mathrm{RA}}\left(t\right)=K\frac{1}{\sigma \sqrt{2\pi }}e^{\frac{-{\left(t-\mu \right)}^{2}}{2\sigma^{2}}}\;,$$

where $\frac{1}{\sigma \sqrt{2\pi }}e^{\frac{-{\left(t-\mu \right)}^{2}}{2\sigma^{2}}}$ is a Gaussian function centred on μ. Fitting to the experimental curve in ([30], Figure 4), we chose μ=30 min that is the time of maximal appearance rate, and σ=35 to obtain ∼70% of the maximum rate at t=0. The value of the parameter *K* is determined by the actual quantity of glucose fed to the rats, such that all the glucose has to be absorbed over the [0,+∞[ time interval. Let $m_{\mathrm{Glc}}$ be the mass of glucose fed to the rats (see Table 2 for the chosen rat body mass in the simulations at PND21, -26, and -60 with respect to the measurement in [17,18]) and $V_{Blood}$ the rat blood volume (see Table 2) as given in [31]. Then, given an administrated concentration of glucose $m_{\mathrm{Glc}}/V_{Blood}$, the value of *K* (Table 2) is the solution of the following equation:

(3) $$\int_{0}^{+\infty }K\frac{1}{\sigma \sqrt{2\pi }}e^{\frac{-{\left(t-\mu \right)}^{2}}{2\sigma^{2}}}\mathrm{dt}=\frac{m_{\mathrm{Glc}}}{V_{Blood}}\;.$$

Upon secretagogue (here glucose) stimulation, insulin release by β-cells occurs in two overlapping phases [32]. The first phase, hereafter called ‘fast’, corresponds to the mobilization of granules belonging to the ‘readily releasable pool’ (RRP) by fusion to the plasma membrane and quasi-immediate excretion: it lasts for a few minutes. The second (‘slow’) phase lasts longer because it involves more complex phenomena such as trafficking to the surface of more deeply stored granules, and production and maturation of new insulin granules up to increased insulin transcription and synthesis to replenish the RRP. The duration of this second phase of the order of 1–2 h allows the organism to start responding to the insulin increase by the uptake of glucose in processing organs such as liver, muscles, and adipose tissues, and parallel insulin clearance by the liver. Measuring glucose clearance in the circulation over time thus integrates all these phenomena and probes the efficiency of insulin secretion by islets of Langerhans in response to glucose and the insulin sensitivity of glucose processing tissues. Of note, the first and second phases of insulin production cannot be distinguished in OGTT as delays in glucose levels in the circulation depend on intestinal absorption. The MINMOD model correctly simulates the slow phase of insulin production, but not the fast one outside of the IVGTT experimental context. To address this problem, we added a state variable representing the insulin already present and ready to be released in the blood circulation. Finally, the adapted MINMOD model is given by Equation (4).

(4) $$\begin{array}{cc} \dot{G} & =-p_{1}\left(G\left(t\right)-G_{b}\right)-r_{Cd}\;X\left(t\right)G\left(t\right)+G_{\mathrm{RA}}\left(t\right)\\ \dot{X} & =-p_{2}X\left(t\right)+p_{3}\left(I\left(t\right)-I_{b}\right)\\ \dot{I} & =-n\;I\left(t\right)+\gamma \;\left(G\left(t\right)-h\right)\;t+p_{4}I_{s}\left(t\right)\\ {\dot{I}}_{s} & =-p_{4}I_{s}\left(t\right) \end{array}$$

We recall that, in the ODE system in Equation (4), G is the glucose concentration in circulating blood, X is the rate of glucose withdrawal by muscles and adipocytes due to insulin, I is the insulin concentration in circulating blood, and $I_{s}$ is the insulin concentration stored in the β-cells and ready to be released. Here, we did not model the C-peptide concentration nor the liver production or absorption of glucose unlike in the more detailed model of [29].

The parameters $p_{1},p_{2},p_{3},n,\gamma ,G_{b},I_{b}$, and *h* are original parameters of the MINMOD model. However, they were refitted in our experimental context. We refer to Section 2.2 for a more detailed introduction to the original MINMOD model. In addition to the parameters of the original MINMOD model, we added, for our purpose of modeling OGTT and cadmium impact, three additional parameters. The flux associated with glucose intake is modeled by $G_{\mathrm{RA}}\left(t\right)$ in Equation (2). The efficiency of the rate of glucose withdrawal X(t) is modeled by $r_{Cd}$. This parameter $r_{Cd}$ was always taken equal to 1.0 in the simulations corresponding to the data of the control group. Finally, the parameter $p_{4}$ models the secretion rate of the readily releasable pool of insulin modeled by the variable $I_{s}\left(t\right)$.

The datasets were obtained from three groups of pups [18], namely control, Cd1, and Cd2 born from female rats with background, medium, and relatively high cadmium burden, respectively, still all corresponding to low exposure doses [18]. OGTT were performed on pups at weaning (21 days after birth, that is PND21), a few days later after shifting on a regular non contaminated chow (PND26), and pcorfive weeks later (PND60). To estimate the goodness of fit of a given simulation compared to the experimental data, we used the root of the weighted least squares error:

(5) $$\varepsilon \left(k\right)=\sqrt{\sum_{i}W_{i}{\left(x_{exp,i}-x_{simu}\left(t_{i},k\right)\right)}^{2}}\;,$$

where k is a parameter set, $x_{exp,i}$ are the mean values associated to an experimental dataset (see Table 1 for its corresponding population), and $x_{simu}\left(t,k\right)$ its associated simulation of the OGTT. The weight $W_{i}$ is determined by the equation:

$$W_{i}=\frac{1}{\nu_{i}^{2}\left(\sum_{i}x_{exp,i}^{2}\right)}\;,$$

where $\nu_{i}^{2}$ are the variance to the mean associated to the *i*th data point.

It follows that, for a given parameter set k, the lower the fitting error ε(k), the better the fitting of the mean of the experimental data. When fitting the experimental results, this implies a bias in favor of the mean values of the data points as the error ε(k) is 0 if the simulation goes through all mean points. Let us remark that the fitting error will decrease when the variances $\nu_{i}^{2}$ increase: an experimental point is easier to fit when its experimental uncertainty increases. This adjustment allows giving more importance to the fitting of points which are experimentally in close positions as they will be the ones which really matter in the decrease of ε(k). Here, the uncertainty on the experimental results not only comes from the precision of the measurements, but also, and mainly, from the individual variations within groups.

**Figure 3:**
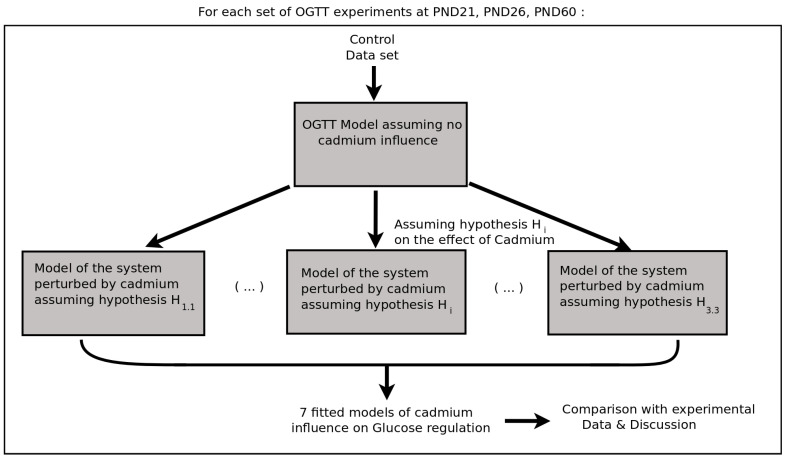
Modeling method to test hypotheses on the possible targets of cadmium. In the diagram, the index *i* spans hypotheses 1.1–3.3.

It is important to note that the error is *specific* to a dataset, and that errors associated to two different datasets cannot be compared. Only comparing the effect of two parameter sets to mean data points (and associated variances) corresponding to a given group (control, Cd1, or Cd2) at a given period (PND21, -26, or -60) makes sense. In Figure 3, we provide a schematic representation of our method to test the possible target of cadmium at PND21, PND26, and PND60, with respect to both our OGTT modeling and the experimental data.

### 3.2. Parameter Analysis

The parameters were searched in the intervals proposed in [21], bloated by one order of magnitude, when possible. The initial value of the concentration of readily releasable insulin was fitted for the control group at PND21 and kept the same for the other experiments. The initial concentration of fasting glucose and insulin in plasma were taken from [18]. We chose to take X(0)=0 as initial condition of the withdrawal rate of glucose: this implies a lack of regulation effect at t = 0. To relax the parameter search, when fitting the parameters to the datasets corresponding to groups Cd1 and Cd2, we only considered a few hypotheses on the evolution of the parameters, starting from the ones fitting the control group, by only altering one parameter at a time.

We considered the following hypotheses for the possible effects of cadmium of plasma glucose regulation:

**Hypothesis** **1.** *Modification of the sensitivity of insulin sensitive tissues.*

- ***Hypothesis 1.1:*** *$r_{Cd}$ varies: this shows the effect of cadmium on the glucose removal by the tissues. If $r_{Cd}<1$, then the system has developed insulin resistance.*
- ***Hypothesis 1.2:*** *$p_{3}$ varies: this represents the effect of insulin on the rate of glucose withdrawal from the circulation.*
- ***Hypothesis 1.3:*** *$p_{2}$ varies: this affects the decrease rate of X(t), which is the glucose withdrawal rate.*

**Hypothesis** **2.** *n varies: this models an effect on insulin degradation.*

**Hypothesis** **3.** *Modification of the insulin release rate.*

- ***Hypothesis 3.1:*** *γ varies: this models an evolution of the insulin release rate, in response to glucose, in the slow phase of insulin production.*
- ***Hypothesis 3.2:*** *$p_{4}$ varies: this models the stored insulin release rate.*
- ***Hypothesis 3.3:*** *h varies: this affects the glucose response threshold.*

The hypotheses on the effect of cadmium on the regulation of circulating glucose were tested in the following manner. We first looked for a starting parameter set $k_{ctrl}$ minimizing Equation (5) for the experimental results of the control group. Then, for experimental results of group Cd1 (respectively Cd2), we first took a hypothesis $H_{i}$ and we refitted a parameter set $k_{H_{i}}$ by varying only one parameter at a time as defined above; the values of the other parameters were taken equal to the ones in the $k_{ctrl}$ parameter set.

#### 3.2.1. Results at PND21 (Weaning)

**Figure 4:**
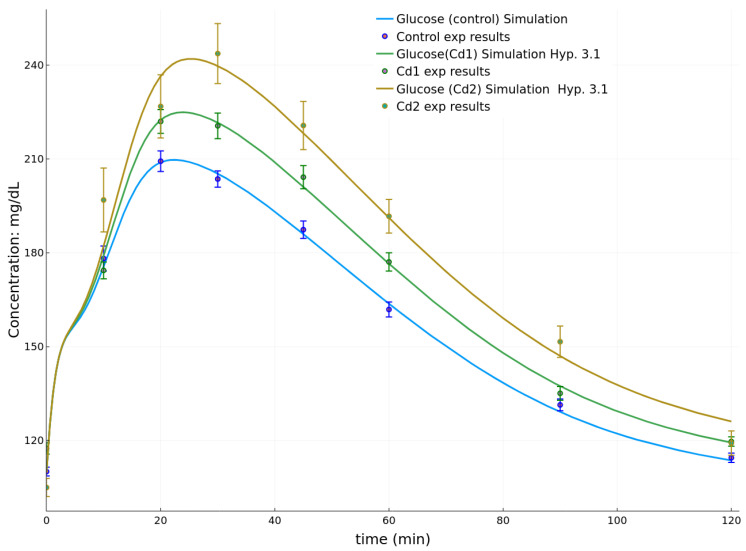
Simulations of the OGTT at PND21 for the control group, Cd1 and Cd2 groups.

For 21-day-old pups at weaning, the goodness of fit for each hypothesis and associated to group Cd1 dataset are shown in Table 3. Considering the initial conditions from Table 4, the associated best parameter sets are given in Table 5. The simulation corresponding to the best parameter fits are shown in Figure 4. It should be reminded here that the experimental points reported in Figure 4 showed significant differences between the animal groups for the areas under the arbitrary drawn curves (AUC) in previously published data [17,18]. Here, the goodness of fit associated to the control group dataset is 0.00311 (Table 6). The best fits for group Cd1 are obtained considering Hypothesis 3.1, i.e., decreased response of β-cells to glucose in the slow phase of insulin production. For the experiment associated to group Cd2 at PND21, the best fit is also obtained for Hypothesis 3.1 and yields a goodness of 0.00433: it shows a continuous decrease of γ as a function of increased cadmium burden of the dams (Table 5).

#### 3.2.2. Results at PND26

Concerning the experiments at PND26, after weaning at PND21 the pups shifted from a milk-based, i.e., lipid-dominated, diet to conventional rodent chow which is rich in carbohydrates. This change of diet induces important changes in the regulation mechanism: this translates into considerable changes of the parameter set fitting the control group dataset. In addition, the three groups of young animals were no longer differently exposed to cadmium after weaning, and the AUC as previously reported [18] did not show any statistically significant difference.

**Figure 5:**
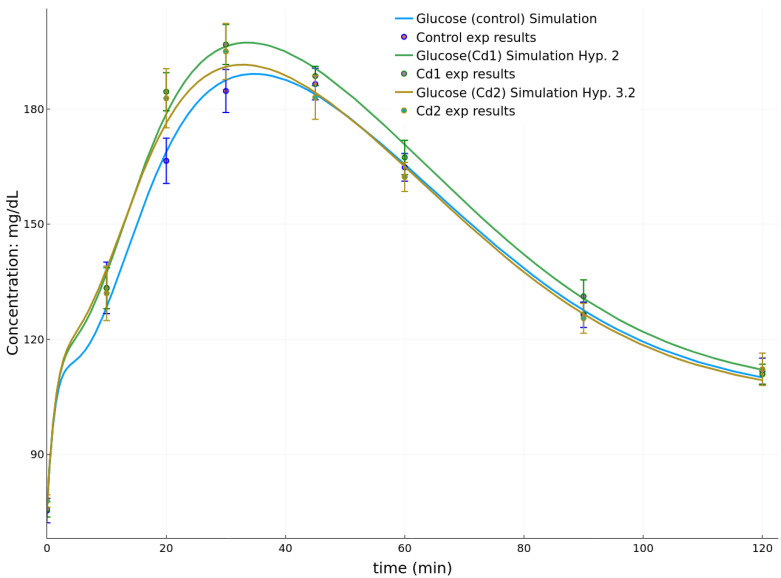
Simulations of the OGTT at PND26 for the control group, Cd1 and Cd2 groups.

The initial conditions at PND26 are given in Table 7. The goodness of fit for each hypothesis on groups Cd1 and Cd2 are given in Table 8. The associated best parameters sets are given in Table 9. The simulation associated to the best fits are shown in Figure 5. The goodness of fit associated to the control group dataset is 0.00315 (see Table 6). We note that, unlike at PND21, there is no clear hypothesis that appears more likely than the others. Indeed, for group Cd1, although Hypothesis 2 yielded the best results, there is only a small difference with Hypotheses 1.1–1.3: thus, the present data cannot discriminate between changes of the insulin sensitivity of muscle and adipose tissue or of the insulin degradation rate.

For group Cd2, we first observe that the higher variance on the experimental results leads to a lower error associated to the control parameter set (denoted no Hyp. in Table 8). In addition, even though Hypothesis 3.2 (reduction in the RRP release rate) yields the best goodness of fit, there is no clear distinction with the other hypotheses because of the increased variance. It follows that the previous interpretation given for group Cd1, namely that of a cadmium effect on the sensitivity of tissues to insulin or the hormone turnover, may still be valid.

#### 3.2.3. Results at PND60

**Figure 6:**
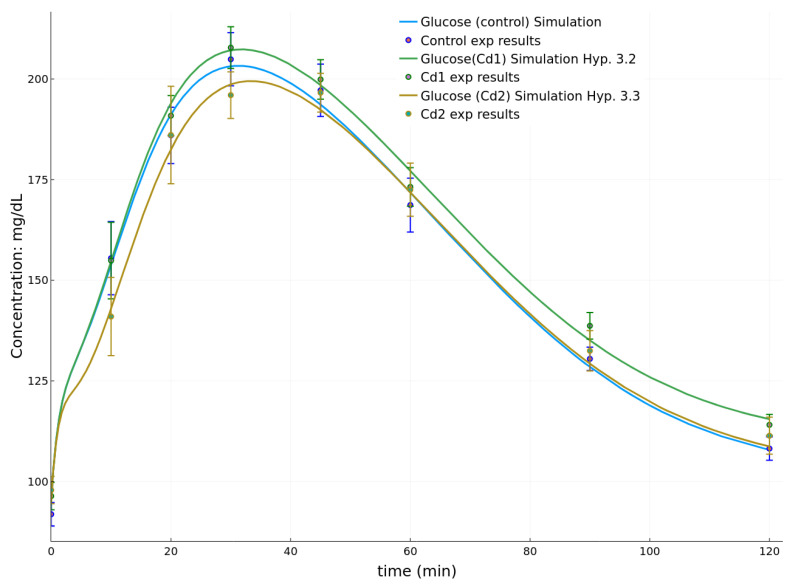
Simulations of the OGTT at PND60 for the control group and groups Cd1 and Cd2.

Finally, considering the initial conditions from Table 10, the parameter set fitted to the datasets at PND60 are given in Table 11. The goodness of fit of the control group dataset is 0.00251 (see Table 6). It can be observed in Figure 6 that group Cd1 from mothers with medium cadmium burden and group Cd2 from the most intoxicated mothers show opposite behaviors with respect to the control curve. Whereas group Cd1 behaved in a similar fashion to the previous results at PND26 with a higher glucose peak than the control glucose response, the results of group Cd2 are characterized by a lower glucose peak.

In Table 12, we notice for group Cd1 that except Hypotheses 3.1 and 3.3, none of the other hypotheses have improved significantly the goodness of fit. However, Hypotheses 3.1 and 3.3 are not among the ones providing an important improvement of the fit for the results associated to group Cd1 at PND26. This raises the possibility of a delayed effect of cadmium on the function of β–cells after exposure during the perinatal period, in agreement with the variations of the C-peptide observed with the same animals [18].

For the results associated with group Cd2, the first three data points are below their control equivalent (see Figure 6). That means that, at PND60, group Cd2 (the one born of mothers with the highest cadmium burden) has a faster removal of glucose from the circulation in the first 40 min. In the simulation, it corresponds to an increase of the parameter p4 (Hypothesis 3.2) which best fits this evolution. All the other hypotheses demonstrate only marginal, or no, improvements.

## 4. Discussion

As shown in the previous section, we tested through simulations various hypotheses that may explain the cadmium influence on the glucose regulation mechanism by insulin. To this aim, we started from a model reproducing correctly the control experiment with pups from mothers that were not intentionally exposed to cadmium. Then, we tested each hypothesis by varying a single parameter until it best fitted the dataset associated with the studied group of cadmium exposed pups. For this reason, we can only provide insight on the effect of one mechanism at a time.

An apparent weakness of our approach might be the risk of overfitting a single curve with at least 10 parameters for four variables. However, once the initial conditions have been set, the procedure aims at testing a series of hypotheses by varying a single parameter at a time to optimize fitting of a complex curve. This way a clear trend may appear, as for the probed data at weaning (PND21), or not. Thus, it is possible to safely avoid over-interpreting the simulations. The main practical interest of the modeling effort is to readily help sorting out the most relevant effects of a perturbation of glucose homeostasis.

For the experiments at PND21, we observed that, for both groups Cd1 and Cd2, it is the hypothesis of a decreased response to glucose during the slow phase of production that best represents the changes between the control group and the more exposed groups. On the experimental datasets, this is noticeable by an increased plasma glucose concentration over the [30,90] min interval, which previously led to increased AUC [18]. The fitted simulations under Hypothesis 3.1 show a good fitting especially on this interval.

However, a few noticeable points of the experimental datasets are not correctly represented by the simulation with Hypothesis 3.1. In particular for group Cd2, the points at 10 and 120 min are not correctly fitted by this single assumption on γ. The experimental point at 10 min, being higher than the simulation, suggests an additional effect in the first phase of insulin secretion. This is represented in our model by the parameter $p_{4}$. However, this parameter should be affected together with γ, as Table 3 shows that Hypothesis 3.2 ($p_{4}$ varies alone) does not correctly fit the data. Insights on the differences for the point at 120 min are more difficult to explain as they can be due to multiple reasons such as: a too coarse approximation of the glucose rate of appearance at 120 min, an effect on a mechanism not represented in the model such as gluconeogenesis, the role of other hormones than insulin, or a competition between multiple opposing effects that are not represented by our single hypothesis. In any case, the present results can be compared with the observations made in [18]. Few biochemical parameters were found to vary and consequently it was not possible to provide a robust explanation to the changes of the OGTT results and associated AUC. Here, simulations of these curves point to the decreased sensitivity of β-cells to glucose as the underlying factor. This decreased sensitivity is proportional to the dams’ cadmium burden (Table 4), and it influences lipid metabolism [18]. The result applies to pups at weaning, i.e., at an age when the endocrine pancreas has yet to fully mature.

The experimental results at PND26 and PND60 represent the evolution of the three groups without additional exposure to cadmium through feeding beyond weaning: they show the lasting effect on the metabolism of a previous cadmium exposure, even though the AUC derived from OGTT were no longer significantly different between animal groups [18].

At PND26, we can already observe a slight behavioral difference with the previous datasets at PND21: whereas at PND21 the glucose concentration of Cd2 is always significantly above Cd1 and control ([18], Figure 5), at PND26, all groups share similar results. Looking to the mean values, one observes that the dataset of group Cd2 is closer to control than Cd1, which is confirmed in the simulation by the lower fitting error for Cd2 compared to Cd1 in absence of hypothesis (see Table 8). Even with this bias in favor of mean points, it is hard to reach any strong conclusion. The experimental results of group Cd1 are best fitted under the hypotheses of a reduced sensitivity to insulin of the glucose withdrawing tissues, or a faster degradation rate of insulin (Hypotheses 1.1–2). The experimental results of group Cd2 are so close to the control experimental results that no hypothesis seems much better than another.

Finally, at PND60, the differences among the three groups are not statistically significant ([18], Figure 9). This is witnessed by the small error associated with the entry without hypothesis in Table 12. When looking to the mean values, it is surprising to note that they are lower for group Cd2, and larger than control for Cd1. The best fit for group Cd1 are Hypotheses 3.1 and 3.3 that are modeling a negative effect on the slow phase of insulin production. The best fit for group Cd2 is the Hypothesis 3.2 modeling a positive effect on the first phase of insulin production.

## 5. Conclusions

In this work, we propose an extension of the MINMOD model [21] to simulate the circulating glucose evolution during OGTT. We fitted OGTT experimental results performed on rats at different ages after cadmium exposure through their mothers. Using these fitted models, we checked various different hypotheses on the effect of cadmium on the glucose response, by comparing the implementation of these hypotheses in the model to the experimental OGTT results for rats exposed to cadmium.

These simulations indicate that dams’ exposure to cadmium negatively affects the slow phase of insulin release in response to glucose in pups at weaning. For the other experimental results at PND26 and PND60, there are no significant differences, yet the modeling approach agrees with the proposed long-lasting effects of cadmium in young animals, long after the indirect exposure via their mothers has ceased.

The results of this study may be extended by the development of a more complex model to better approximate the glucose appearance rate in the context of an OGTT, as well as the mechanism affected by Hypothesis 3.1. For this purpose, the comprehensive modeling of metabolism leading to insulin secretion [33] should be included when relevant experimental data become available. In this paper, we only propose a single fitting parameter set, but probing more complete datasets with more exhaustive and costly methods should be performed in order to further assert our conclusions. Finally, this model has only fitted the experimental glucose response, but not the evolution of insulin plasma concentration over time during OGTT in young rats: combining such more extensive datasets would be important to develop the modeling approach to be used in a more quantitative manner.

## Figures and Tables

**Table 1:** Number of animals in the control, Cd1 and Cd2 groups at PND21, PND26, and PND60.

	PND21	PND26	PND60
Control	48	13	12
Cd1	38	18	17
Cd2	35	10	12

**Table 2:** Values of body mass, blood volume, and parameter *K* at PND21, -26 and -60.

	PND21	PND26	PND60	Unit
Body mass	45	65	205	g
Blood volume	0.03	0.04	0.128	dL
*K*	3729	4040	3982	mg/(dL·min)

**Table 3:** Goodness of fit from Equation (5) of each hypothesis applied to the Cd1 and Cd2 datasets at PND21. The symbol − denotes a value no better than the control parameter set.

Hypothesis	Cd1	Cd2
**No Hyp.**	0.0188	0.0144
**Hyp 1.1**	0.0144	0.00820
**Hyp 1.2**	0.0144	0.00821
**Hyp 1.3**	0.0140	0.00789
**Hyp 2**	0.0145	0.00852
**Hyp 3.1**	0.00566	0.00433
**Hyp 3.2**	−	0.0137
**Hyp 3.3**	0.0102	0.00740

**Table 4:** Initial condition determined for the control group at PND21. These initial conditions are conserved for groups Cd1 and Cd2. Note that 1 U = 0.0347 mg of insulin.

Variable	Value	Unit
G(0)	110.0	mg/dL
X(0)	0.0	min${}^{-1}$
I(0)	16.0	nU/dL
$I_{s}\left(0\right)$	5950.0	nU/dL

**Table 5:** Parameters values fitted for the control group as well as groups Cd1 and Cd2 at PND21 (considering Hypothesis 3.1 for both groups).

Parameters	Ctrl	Cd1	Cd2	Unit
$p_{1}$	0.01	−	−	min${}^{-1}$
$G_{b}$	100.0	−	−	mg/dL
$p_{2}$	0.56	−	−	min${}^{-1}$
$p_{3}$	0.0155	−	−	(dL/nU)min${}^{-2}$
$I_{b}$	10.0	−	−	nU/dL
*n*	10.53	−	−	min${}^{-1}$
γ	0.0310	0.0258	0.0215	(nU/dL)min${}^{-2}$
*h*	85.0	−	−	mg/dL
$p_{4}$	0.033	−	−	min${}^{-1}$
$r_{Cd}$	1.0	−	−	N.U.

**Table 6:** Goodness of fit from Equation (5) for each dataset of the control group at PND21, -26 and -60.

Control Groups	Goodness of Fit
PND21	0.00311
PND26	0.00315
PND60	0.00251

**Table 7:** Initial condition determined for the control group and groups Cd1 and Cd2 at PND26.

Variable	Value	Unit
G(0)	76.0	mg/dL
X(0)	0.0	min${}^{-1}$
I(0)	34.0	nU/dL
$I_{s}\left(0\right)$	5950.0	nU/dL

**Table 8:** Goodness of fit from Equation (5) of each hypothesis applied to the datasets of groups Cd1 and Cd2 at PND26.

Hypothesis	Cd1	Cd2
**No Hyp.**	0.00972	0.00600
**Hyp 1.1**	0.00411	0.00444
**Hyp 1.2**	0.00410	0.00435
**Hyp 1.3**	0.00398	0.00434
**Hyp 2**	0.00395	0.00442
**Hyp 3.1**	0.00762	0.00584
**Hyp 3.2**	0.00638	0.00388
**Hyp 3.3**	0.00819	0.00465

**Table 9:** Parameters values fitted for the control group as well as groups Cd1 and Cd2 at PND26 (considering Hypothesis 3.2 for Cd2 and Hypothesis 2 for Cd1).

Parameters	Ctrl	Cd1	Cd2	Unit
$p_{1}$	0.01	−	−	min${}^{-1}$
$G_{b}$	100.0	−	−	mg/dL
$p_{2}$	0.61	−	−	min${}^{-1}$
$p_{3}$	0.0245	−	−	(dL/nU)min${}^{-2}$
$I_{b}$	05.0	−	−	nU/dL
*n*	09.44	9.93	−	min${}^{-1}$
γ	0.0110	−	−	(nU/dL)min${}^{-2}$
*h*	79.0	−	−	mg/dL
$p_{4}$	0.0215	−	0.201	min${}^{-1}$
$r_{Cd}$	1.0	−	−	N.U.

**Table 10:** Initial condition determined for the control group and groups Cd1 and Cd2 at PND60.

Variable	Value	Unit
G(0)	95.0	mg/dL
X(0)	0.0	min${}^{-1}$
I(0)	34.0	nU/dL
$I_{s}\left(0\right)$	5950.0	nU/dL

**Table 11:** Parameter values fitted for the control group as well as groups Cd1 and Cd2 at PND60 (considering Hypothesis 3.3 for Cd2 and Hypothesis 3.2 for Cd1).

Parameters	Ctrl	Cd1	Cd2	Unit
$p_{1}$	0.01	−	−	min${}^{-1}$
$G_{b}$	100.0	−	−	mg/dL
$p_{2}$	0.79	−	−	min${}^{-1}$
$p_{3}$	0.0335	−	−	(dL/nU)min${}^{-2}$
$I_{b}$	06.0	−	−	nU/dL
*n*	8.35	−	−	min${}^{-1}$
γ	0.0078	−	−	(nU/dL)min${}^{-2}$
*h*	65.0	73.0	−	mg/dL
$p_{4}$	0.0170	−	0.0180	min${}^{-1}$
$r_{Cd}$	1.0	−	−	N.U.

**Table 12:** Goodness of fit from Equation (5) of each hypothesis applied to the datasets of groups Cd1 and Cd2 at PND60.

Hypothesis	Cd1	Cd2
**No Hyp.**	0.00440	0.00403
**Hyp 1.1**	0.00395	0.00304
**Hyp 1.2**	0.00395	0.00304
**Hyp 1.3**	0.00396	0.00312
**Hyp 2**	0.00374	0.00325
**Hyp 3.1**	0.00267	−
**Hyp 3.2**	0.00439	0.00194
**Hyp 3.3**	0.00204	0.00402

## Abbreviations

The following abbreviations are used in this manuscript:

PND21	Post-Natal Day 21
PND26	Post-Natal Day 26
PND60	Post-Natal Day 60
RRP	Readily Releasable Pool
OGTT	Oral Glucose Tolerance Test
AUC	Area Under the Curve
MINMOD	Minimal Model

## References

[1] Hannon T.S., Kahn S.E., Utzschneider K.M., Buchanan T.A., Nadeau K.J., Zeitler P.S., Ehrmann D.A., Arslanian S.A., Caprio S., Edelstein S.L. (2018). Review of methods for measuring *β*-cell function: Design considerations from the Restoring Insulin Secretion (RISE) Consortium. Diabetes Obes. Metab..

[2] Schwartz G.G., Il’yasova D., Ivanova A. (2003). Urinary cadmium, impaired fasting glucose, and diabetes in the NHANES III. Diabetes Care.

[3] Barregard L., Bergström G., Fagerberg B. (2013). Cadmium exposure in relation to insulin production, insulin sensitivity and type 2 diabetes: A cross-sectional and prospective study in women. Environ. Res..

[4] Guo F.F., Hu Z.Y., Li B.Y., Qin L.Q., Fu C., Yu H., Zhang Z.L. (2019). Evaluation of the association between urinary cadmium levels below threshold limits and the risk of diabetes mellitus: A dose-response meta-analysis. Environ. Sci. Pollut. Res. Int..

[5] Kuo C.C., Moon K., Thayer K.A., Navas-Acien A. (2013). Environmental chemicals and type 2 diabetes: An updated systematic review of the epidemiologic evidence. Curr. Diab. Rep..

[6] Li Y., Zhang Y., Wang W., Wu Y. (2017). Association of urinary cadmium with risk of diabetes: A meta-analysis. Environ. Sci. Pollut. Res. Int..

[7] Moon S.S. (2013). Association of lead, mercury and cadmium with diabetes in the Korean population: The Korea National Health and Nutrition Examination Survey (KNHANES) 2009–2010. Diabet Med..

[8] Noor N., Zong G., Seely E.W., Weisskopf M., James-Todd T. (2018). Urinary cadmium concentrations and metabolic syndrome in US adults: The National Health and Nutrition Examination Survey 2001–2014. Environ. Int..

[9] Tinkov A.A., Filippini T., Ajsuvakova O.P., Aaseth J., Gluhcheva Y.G., Ivanova J.M., Bjørklund G., Skalnaya M.G., Gatiatulina E.R., Popova E.V. (2017). The role of cadmium in obesity and diabetes. Sci. Total Environ..

[10] Wallia A., Allen N.B., Badon S., El Muayed M. (2014). Association between urinary cadmium levels and prediabetes in the NHANES 2005–2010 population. Int. J. Hyg. Environ. Health.

[11] Rahman A., Kumarathasan P., Gomes J. (2016). Infant and mother related outcomes from exposure to metals with endocrine disrupting properties during pregnancy. Sci. Total Environ..

[12] Shapiro G., Dodds L., Arbuckle T., Ashley-Martin J., Fraser W., Fisher M., Taback S., Keely E., Bouchard M., Monnier P. (2015). Exposure to phthalates, bisphenol A and metals in pregnancy and the association with impaired glucose tolerance and gestational diabetes mellitus: The MIREC study. Environ. Int..

[13] Huang C.C., Kuo C.Y., Yang C.Y., Liu J.M., Hsu R.J., Lee K.I., Su C.C., Wu C.C., Lin C.T., Liu S.H. (2019). Cadmium exposure induces pancreatic *β*-cell death via a Ca2+-triggered JNK/CHOP-related apoptotic signaling pathway. Toxicology.

[14] Li X., Li M., Xu J., Xiao W., Zhang Z. (2019). Decreased insulin secretion but unchanged glucose homeostasis in cadmium-exposed male C57BL/6 mice. J. Toxicol..

[15] Jacquet A., Ounnas F., Lenon M., Arnaud J., Demeilliers C., Moulis J.M. (2016). Chronic exposure to low-level cadmium in diabetes: Role of oxidative stress and comparison with polychlorinated biphenyls. Curr. Drug Targets.

[16] Barry E., Roberts S., Oke J., Vijayaraghavan S., Normansell R., Greenhalgh T. (2017). Efficacy and effectiveness of screen and treat policies in prevention of type 2 diabetes: Systematic review and meta-analysis of screening tests and interventions. BMJ.

[17] Jacquet A. (2017). Conséquences d’une Exposition Chronique à des Doses Modérées de Cadmium sur le Métabolisme du Glucose de Rats à Différents Stades de la vie. Ph.D. Thesis.

[18] Jacquet A., Barbeau D., Arnaud J., Hijazi S., Hazane-Puch F., Lamarche F., Quiclet C., Couturier K., Fontaine E., Moulis J.M. (2019). Impact of maternal low-level cadmium exposure on glucose and lipid metabolism of the litter at different ages after weaning. Chemosphere.

[19] Jacquet A., Arnaud J., Hininger-Favier I., Hazane-Puch F., Couturier K., Lénon M., Lamarche F., Ounnas F., Fontaine E., Moulis J.M. (2018). Impact of chronic and low cadmium exposure of rats: Sex specific disruption of glucose metabolism. Chemosphere.

[20] Pacini G., Bergman R.N. (1986). MINMOD: A computer program to calculate insulin sensitivity and pancreatic responsivity from the frequently sampled intravenous glucose tolerance test. Comput. Methods Prog. Biomed..

[21] Nittala A., Ghosh S., Stefanovski D., Bergman R., Wang X. (2006). Dimensional analysis of MINMOD leads to definition of the disposition index of glucose regulation and improved simulation algorithm. Biomed. Eng. Online.

[22] Rackauckas C., Nie Q. (2017). DifferentialEquations.jl—A performant and feature-rich ecosystem for solving differential equations in Julia. J. Open Res. Softw..

[23] Kang H., Han K., Choi M. (2012). Mathematical model for glucose regulation in the whole-body system. Islets.

[24] Breda E., Cavaghan M.K., Toffolo G., Polonsky K.S., Cobelli C. (2001). Oral glucose tolerance test minimal model indexes of *β*-cell function and insulin sensitivity. Diabetes.

[25] Breda E., Toffolo G., Polonsky K.S., Cobelli C. (2002). Insulin release in impaired glucose tolerance: Oral minimal model predicts normal sensitivity to glucose but defective response times. Diabetes.

[26] Dalla Man C., Campioni M., Polonsky K.S., Basu R., Rizza R.A., Toffolo G., Cobelli C. (2005). Two-hour seven-sample oral glucose tolerance test and meal protocol. Diabetes.

[27] Dalla Man C., Caumo A., Basu R., Rizza R., Toffolo G., Cobelli C. (2005). Measurement of selective effect of insulin on glucose disposal from labeled glucose oral test minimal model. Am. J. Physiol. Endocrinol. Metab..

[28] Dalla Man C., Rizza R.A., Cobelli C. (2007). Meal simulation model of the glucose-insulin system. IEEE Trans. Biomed. Eng..

[29] Cobelli C., Dalla Man C., Toffolo G., Basu R., Vella A., Rizza R. (2014). The oral minimal model method. Diabetes.

[30] Wielinga P.Y., Wachters-Hagedoorn R.E., Bouter B., van Dijk T.H., Stellaard F., Nieuwenhuizen A.G., Verkade H.J., Scheurink A.J. (2005). Hydroxycitric acid delays intestinal glucose absorption in rats. Am. J. Physiol. Gastrointest. Liver Physiol..

[31] Constable B. (1963). Changes in blood volume and blood picture during the life of the rat and guinea-pig from birth to maturity. J. Physiol..

[32] Henquin J.C., Nenquin M., Stiernet P., Ahren B. (2006). In vivo and in vitro glucose-induced biphasic insulin secretion in the mouse: Pattern and role of cytoplasmic Ca2+ and amplification signals in *β*-cells. Diabetes.

[33] Salvucci M., Neufeld Z., Newsholme P. (2013). Mathematical model of metabolism and electrophysiology of amino acid and glucose stimulated insulin secretion: In vitro validation using a *β*-cell line. PLoS ONE.

